# Knowledge embedded wind tunnel typical pressure vessel design optimization method research

**DOI:** 10.1371/journal.pone.0350925

**Published:** 2026-06-09

**Authors:** Yisheng Yang, Sijie Yan, Qiang Xie, Bowen Liu, Xiqiang Yan, Zeyuan Yang, Han Ding

**Affiliations:** 1 State Key Lab of Intelligent Manufacturing Equipment and Technology, Huazhong University of Science and Technology, Wuhan, China; 2 China Aerodynamics Research and Development Center, Mianyang, China; TU Dublin Blanchardstown Campus: Technological University Dublin - Blanchardstown Campus, IRELAND

## Abstract

Wind tunnels serve as an essential infrastructure for aerodynamic research and aerospace vehicle development, and pressure vessels are the most important type of structure in transonic and supersonic wind tunnels. Conventional structural design methodologies exhibit critical limitations including over-reliance on empirical specifications, computational inefficiency, and excessive conservatism. Although data-driven surrogate-based optimization approaches partially mitigate these issues, their generalizability variable operation condition remains limited. This study proposes a knowledge-embedded hierarchical Kriging (KEHK) framework that synergistically integrates the pressure vessel design specification with adaptive multi-fidelity modeling. The methodology introduces three key innovations: a knowledge-embedded sequential sampling method based on pressure vessel design specification, an adaptive hierarchical Kriging architecture incorporating multi-fidelity training samples, and a novel condition-mapping protocol to enhance cross-scenario generalizability. The experimental validation of a transonic wind tunnel acceleration section demonstrated a 26.2% structural weight reduction while maintaining operational integrity, coupled with a 150 × computational efficiency improvement over conventional finite element analysis for single-iteration simulations. Comparative evaluations revealed the KEHK model’s superior generalization capability, achieving a prediction error of <15% across 0.1–2.0 the operational pressure ranges, significantly outperforming the conventional hierarchical Kriging, BPNN and nonadaptive KEHK variants. These advancements have established the framework as a robust solution for next-generation wind tunnel engineering applications, effectively bridging the gap between the computational efficiency and operational reliability.

## I. Introduction

**Fig 1:**
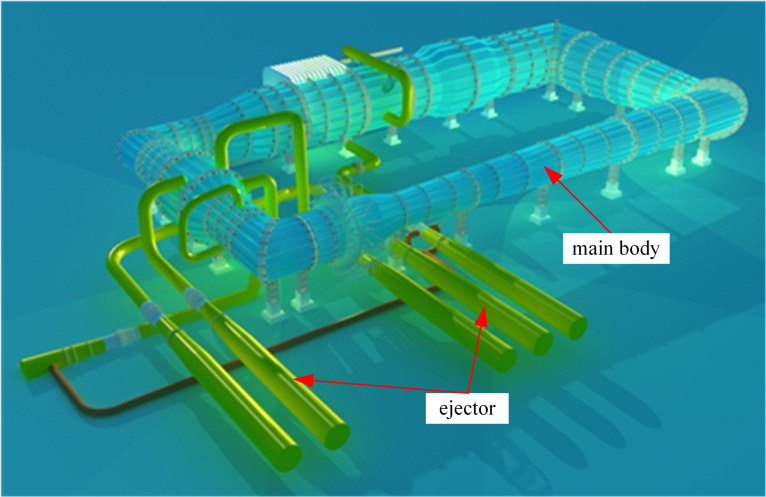
Layout diagram of a semi-recirculating transonic wind tunnel.

Wind tunnels serve as critical infrastructure for aerodynamic research, enabling precise reproduction of flow fields around vehicles through relative motion principles [1]. These controlled environments have essential applications in aerospace vehicle development, structural wind-induced vibration analysis, and renewable energy equipment evaluation. A semi-recirculating transonic wind tunnel layout could be seen in Fig 1, the main body and ejector are both pressure vessel.

In transonic and supersonic wind tunnels, the total operating pressure is a critical parameter for assessing simulation fidelity during test evaluations. Structural design encompasses pressure shell design, overall layout configuration, model support mechanisms, and power systems. Among these, the pressure shell is a central component because it ensures the structural integrity of the wind tunnel while bearing the pressure differential loads between the interior and exterior. The design must adhere to established pressure vessel specifications.

As evidenced by the configurations of leading transonic/supersonic wind tunnels operated by global aerodynamics research institutes (e.g., CARDC, NASA, ONERA, and TsAGI), the most representative pressure-shell structure is the circumferentially stiffened pressure vessel. Thus, developing an adaptive optimization methodology for annular-reinforced structures is essential for advancing intelligent wind tunnel designs.

**Fig 2:**
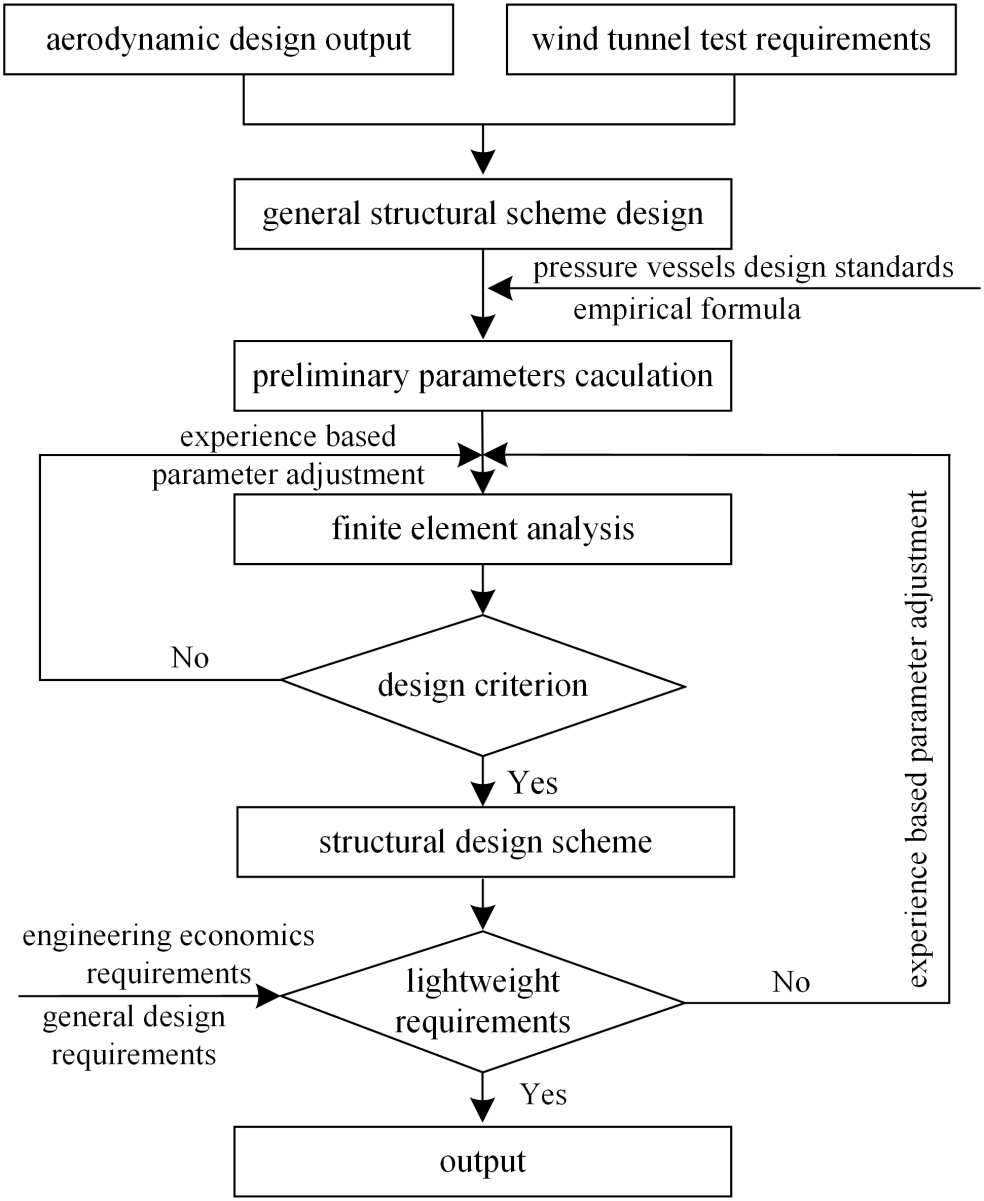
Conventional wind tunnel structural design process.

The conventional wind tunnel pressure vessel design methodology (Fig 2) involves three sequential stages: (1) parameter determination through operational requirement decomposition and aerodynamic profile design; (2) reliability verification via engineering specification compliance checks; and (3) localized optimization employing finite element analysis (FEA) with empirical corrections. Despite ensuring structural integrity, this paradigm has two limitations. First, the safety factor approach frequently produces overly conservative designs that conflict with the modern objectives of optimal strength-to-weight ratio. Second, FEA encounters three inherent challenges in multiparameter co-optimization: mesh dependency, algorithmic nonconvergence, and prohibitive computational costs [2].

Surrogate modeling offers an innovative solution by employing mathematical formulations to approximate complex system responses, effectively addressing the computational inefficiency of conventional approaches [3]. Recent advances have demonstrated diverse applications. Winkler et al. [4] systematically classified machine learning applications in chemical-mechanical planarization across four domains, providing valuable insights for wind tunnel digital systems. Zhou et al. [5] developed a multilevel graph-based surrogate model with enhanced spatial edge weights, which was validated through hot-stamping B-pillar cases, although its applicability to complex 3D geometries requires further verification. Among various techniques, Gaussian-process-based Kriging stands out for structural optimization because of its unbiased estimation and error quantification capabilities [6–11]. The seminal AK-MCS framework proposed by Echard et al. [12] intelligently couples Kriging with Monte Carlo Simulation, utilizing the U-learning function to adaptively sample critical regions, dramatically reducing the required high-fidelity simulations by orders of magnitude for reliability analysis.

Recent developments in multi-fidelity modeling show particular promise: Co-Kriging [13] and hierarchical Kriging (HK) [14] substantially reduce computational costs through low-fidelity samples. While Co-Kriging requires solving covariance matrices, HK establishes multi-fidelity correlations via adaptive response factors, eliminating complex matrix operations. Building on HK, Bu et al. [15] proposed multilevel hierarchical Kriging (MHK) integrating ≥3 fidelity models, achieving 7 dB noise reduction in helicopter blades, although critical relationships between model layers and optimization efficiency remain unexplored.

Structural optimization research revealed distinct methodological trajectories. Yi et al. [16] investigated the sample ratio impacts on the Co-Kriging accuracy for reinforced column shells, while Xu et al. [17] achieved an 11.9% mass reduction in vehicle cabins through Kriging driven optimization. The advent of composite materials has expanded surrogate applications to include manufacturing variables such as fiber orientation angles [18] and mechanical properties such as flexural strength [19,20]. Sudret proposed polynomial-chaos-kriging (PC-kriging) [21], which offers a robust framework for fusing models of varying fidelity and cost. This approach is particularly effective for rare event estimation in high-dimensional problems that are commonly encountered in complex structural systems [22].

**Fig 3:**
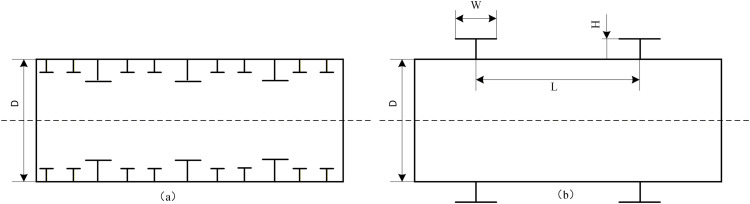
Typical aerospace and wind tunnel structural diagram. (a) Aerospace thin-walled reinforced structure. (b) Wind tunnel external reinforcement configuration.

Current applications predominantly focus on internal mesh-reinforced structures as shown in Fig 3a, which could be widely found in structures subjected to external pressure (e.g., seaprobe and rockets). It is imperative to note that, within the confines of a transonic wind tunnel, both the internal and external pressures can attain considerable heights. To obtain a smooth and continuous internal profile curve, the external reinforcement method is usually adopted in wind tunnel, as shown in Fig 3b. [23] 10% mass reduction in hydraulic tunnels using radial point interpolation, computational intensity remains a barrier.

Existing surrogate optimization research predominantly focuses on data-driven parameter optimization and static load lightweight. While improving cost-efficiency ratios, this approach fundamentally neglects physics-informed constraints and diverse operating conditions, resulting in suboptimal generalization capacity that hinders industrial deployment [24,25].

To address the aforementioned challenges, this study proposes a knowledge-embedded hierarchical Kriging (KEHK) optimization framework that integrates the pressure vessel design specification and HK model to establish an adaptive optimization framework for wind tunnel pressure vessel design under diverse operating conditions. The incorporation of expertise in wind tunnel structural design into the sample generation stage is critical for ensuring design consistency. By constraining the generation of new samples during the adaptive iteration process of the HK model, the framework maintains consistency between the training sample values and the design specifications. Furthermore, standardizing the optimization results according to the engineering material specification series substantially enhances the engineering applicability of the outcomes. A novel parameter conversion method grounded in operational condition mapping was introduced. This method employs prior knowledge constraints to improve the generalization capability under varying operational scenarios, and presents exceptional potential for practical engineering applications.

The remainder of this paper is organized as follows. Section Ⅱ details the proposed knowledge-embedded sequential sampling method, and the KEHK model architecture and adaptive optimization mechanism are subsequently presented. In Section Ⅲ, a validation case regarding the optimization of the structural design of a typical pressure vessel in a transonic wind tunnel is provided. In Section Ⅳ, the impact of sample size on model accuracy and generalizability across operational domains is discussed. Section Ⅴ synthesizes the key findings and discusses prospects for engineering implementation.

### Knowledge-embedded hierarchical kriging optimization framework

#### A. Knowledge-embedded sequential sampling method.

The experimental design process involved the generation of training sample points within the designated design space. Obtaining sufficient model information with a reduced number of sample points is crucial for ensuring the prediction performance of surrogate models [26]. Common methods include uniform sampling, factorial design, central composite design, and Latin hypercube sampling (LHS). LHS is a stratified sampling method that ensures that sampling points can be taken in any part of a given region. Optimal Latin hypercube sampling (OLHS) has been extensively applied owing to its superior space-filling and projection properties [27–28]. While optimal design principles theoretically permit the arbitrary selection of design variables within specified ranges to achieve comprehensive sampling space coverage, practical engineering considerations mandate the implementation of design constraints. These constraints eliminate invalid configurations in which the structural integrity under anticipated loads is compromised. Consequently, nonviable solutions were eliminated from the training datasets, thereby enhancing the practical relevance of the optimization outcomes. It is evident that the static experimental design method is ineffective for addressing such problems. Although mainstream sequential experimental design methods can be continuously updated during the model training process, physical prior knowledge is excluded from the process [29]. To address the issue of transonic and supersonic wind tunnel pressure vessel structure optimization design, a knowledge-embedding sequential sampling method is proposed.

As discussed before, the total operating pressure range of a transonic and supersonic wind tunnel can be positive or negative. This means that the wind tunnel structural design must meet the design specifications for pressure vessels under both internal and external pressures. As outlined in the *Pressure Vessel Design Specification* [30], the initial step in the process is to calculate the thickness of the inner pressure vessel cylinder, as shown in (1).


$$\begin{array}{c} \delta =\frac{PD_{i}}{2{\left[\sigma \right]}^{t}\varphi -P} \end{array}$$ (1)

where δ is the calculated thickness (mm), P *P* is the design pressure (MPa), $D_{i}$ is the internal diameter (mm), [σ]^*t*^ is the permissible stress (185 MPa), and *Φ* is the weld coefficient (assumed as 1).

Equation (1) establishes the minimum permissible thickness of the pressure-vessel shell in the context of the forward design. In accordance with the *Pressure Vessel Design Specification* [30], the calculated stress of the design scheme $\sigma^{t}$ should not exceed the allowable stress ${\left[\sigma \right]}^{t}$ under the corresponding design conditions. Furthermore, the maximum allowable working pressure of the design scheme did not fall short of the design pressure. The mathematical descriptions are delineated in (2) – (5).


$$\begin{array}{c} \sigma^{t}=\frac{p\left(D_{i}+\delta \right)}{2\delta } \end{array}$$ (2)

$$\begin{array}{c} \sigma^{t}\leq {\left[\sigma \right]}^{t} \end{array}$$ (3)

$$\begin{array}{c} \left[p_{w}\right]=\frac{2\delta {\left[\sigma \right]}^{t}\phi }{D_{i}+\delta } \end{array}$$ (4)

$$\begin{array}{c} \left[p_{w}\right]\geq P \end{array}$$ (5)

In the context of external pressure, it is imperative to ensure that the maximum allowable external pressure [p] does not exceed the design pressure. This principle is particularly relevant in the case of pressure vessels such as wind tunnel structures, where the ratio of pipe diameter to wall thickness exceeds 20. In such instances,. The mathematical description is given by (6)–(8):


$$\begin{array}{c} \left[p\right]=\frac{B}{D_{i}/\delta } \end{array}$$ (6)

$$\begin{array}{c} \left[p\right]\leq P \end{array}$$ (7)

$$\begin{array}{c} B=\mathrm{\backslash raisebox}1ex\backslash (2AE^{t}\backslash )\;/\;\mathrm{\backslash raisebox}\mathrm{-1ex}\backslash (3\backslash ) \end{array}$$ (8)

where A and $E^{t}$ represent the external pressure strain coefficient, and the elastic modulus of the material at the design temperature, which can be obtained from relevant charts and tables, respectively, and B represents the external pressure stress coefficient.

Consequently, the wall thickness of the structure should be the maximum value calculated using (1) to (4). As demonstrated in the above equations, the factors influencing minimum wall thickness δ involve design pressure P, internal diameter $D_{i}$, material allowable stress ${\left[\sigma \right]}^{t}$, welding coefficient ϕ, external pressure strain, and stress coefficient A,B. When the inner diameter $D_{i}$ is the input parameter for aerodynamic design, it remains constant throughout the structural design. Under a specific design temperature and chosen material, the allowable stress, welding factor, and external pressure stress coefficient were set as constant. In the final analysis, the design pressure emerged as the predominant factor influencing the wall thickness of the wind tunnel pressure-vessel shell. Moreover, within the operational range of the transonic wind tunnels, the maximum external pressure on the tunnel body does not exceed 1 atm (0.1 MPa). Consequently, the minimum wall thickness determined by (6)–(8) can be embedded in the sampling process as a constant under the design conditions.

To enhance the sample generation quality and ensure design rationality, it is essential to comprehensively consider the design specification constraints outlined in (1)–(8) during the design-variable selection phase. To elaborate further:


$$\begin{array}{c} \delta =S(\delta_{max}-\delta )+\delta \end{array}$$ (9)

where $\delta_{max}$ represents the maximum permissible thickness, measured in millimeters, and S denotes the optimal Latin hypercube sampling parameters.

This methodology not only prevents structural inadequacies under operational loads caused by improper variable combinations but also intrinsically captures the essential relationship between design load parameters and wall thickness requirements through constraint-driven optimization.

In the context of pressure vessel structures stiffened by ribs, it is imperative that the moment of inertia $I_{s}$ at the interface between the rib and effective section of the vessel shell exceeds the moment of inertia necessary I for structural reinforcement.


$$\begin{array}{c} I_{s}\geq I \end{array}$$ (10)

$$\begin{array}{c} I=\frac{{D_{i}}^{\text{2}}L_{s}\left(\delta +A_{s}/L_{s}\right)}{10.9}A \end{array}$$ (11)

$$\begin{array}{c} A_{s}=\frac{\pi \left({\left(D_{i}+2h\right)}^{\text{2}}-{D_{i}}^{\text{2}}\right)}{\text{4}} \end{array}$$ (12)

$$\begin{array}{c} I_{s}=\frac{0.55\sqrt{D_{i}\delta }{D_{i}}^{\text{3}}+\delta {\left(D_{i}+2h\right)}^{\text{3}}}{12} \end{array}$$ (13)

$$\begin{array}{c} B=\frac{PD_{i}}{\delta +\left(A_{s}/L_{s}\right)} \end{array}$$ (14)

where $A_{s}$ represents the area of the section ribs, $L_{s}$ represents half of the distance between two adjacent ribs, and h represents the height of the rib.

As shown in (10)-(12), the moment of inertia of the stiffened section is directly proportional to the shell diameter ($D_{i}$), wall thickness (δ), and rib height (h). The moment of inertia required for the structural reinforcement is proportional to the external pressure stress coefficient (A), shell diameter, wall thickness, and rib spacing ($L_{s}$). As the preceding analysis demonstrates, the contributing factors influencing both moments of inertia for a given design problem under specified conditions, the contributing factors influencing both moments of inertia are solely the external pressure stress coefficient, stiffener height, wall thickness, and rib spacing. According to the pressure vessel design process, the primary parameter to be determined was the shell wall thickness. At this stage, the design moment of inertia for the stiffener cross-section follows a cubic relationship, whereas the minimum moment of inertia maintains an approximately linear relationship with stiffener spacing. (10) can be rewritten as


$$\begin{array}{c} \begin{array}{c} f\left(h,L_{s}\right)=\frac{0.55\sqrt{D\delta }D^{\text{3}}+\delta {\left(D+2h\right)}^{\text{3}}}{12}\\ -\frac{D^{\text{2}}\left(4L_{s}\delta +\pi \left({\left(D+2h\right)}^{\text{2}}-D^{\text{2}}\right)\right)}{43.6}A\geq 0 \end{array} \end{array}$$ (15)

**Fig 4:**
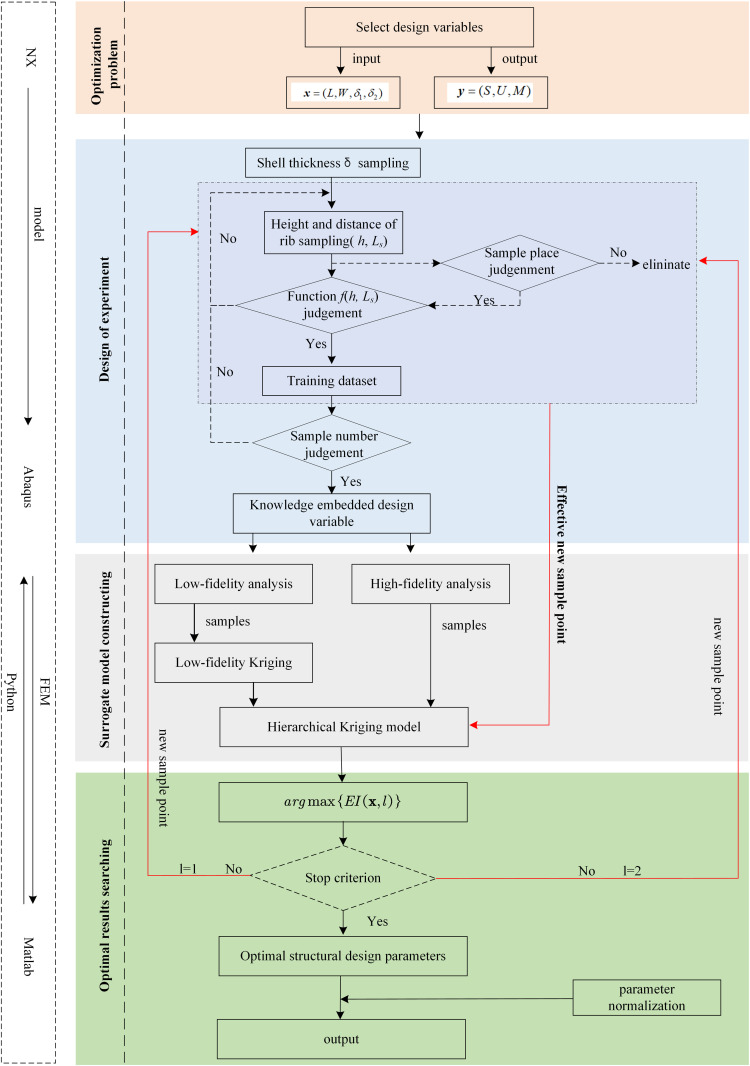
Flowchart of the optimization procedure. Case study on wind tunnel typical pressure vessels design optimization.

As shown in Fig 4, a knowledge-embedded sequential sampling method based on OLHS and pressure vessel design specifications is proposed. The process begins with the initial sampling points obtained using standard OLHS, after which the wall thickness is determined according to (9). Unqualified points are then removed based on (15), with compliant rib distance ($L_{s}$) and height (h) samples stored in an intermediate datasets. OLHS is reapplied to replenish the samples lost in the prior steps. The minimum geometric distance between the initial sampling points is defined as the threshold; if a new sample’s geometric distance to any point in the intermediate datasets falls below this threshold, it is classified as a duplicate and removed. Surviving points were subjected to further qualification checks. These steps were repeated iteratively until a sufficient number of qualified samples were obtained to train the surrogate model.

#### B. Hierarchical kriging model.

The HK surrogate model operates using a dual-phase framework [31]. First, a baseline Kriging model using computationally economical low-fidelity samples ($X_{l},y_{l}$) was established and employed as the model prediction trend. This primary model was then augmented with strategically selected high-fidelity samples ($X_{h},y_{h}$) through an adaptive refinement process, achieving significant computational efficiency gains while maintaining prediction accuracy through systematic error correction. Where, X and y represent the input and output parameters of a datasets that follows a Gaussian distribution, respectively, the superscripts *l* and *h* represent low- and high-fidelity, respectively. The primary Kriging model can be trained as shown in (16) and (17).


$$\begin{array}{c} \left\{ \begin{array}{c} @c@{\hat{y}}_{l}\left(x\right)={\hat{\mu }}_{l}+\overset{T}{\underset{l}{r}}\overset{-1}{\underset{l}{C}}\left(y_{l}-1{\hat{\mu }}_{l}\right)\\ \overset{\text{2}}{\underset{l}{s}}\left(x\right)=\overset{\text{2}}{\underset{l}{\hat{\sigma }}}\left[1-\overset{T}{\underset{l}{r}}\overset{-1}{\underset{l}{C}}r_{l}+\frac{{\left(1-\overset{T}{\underset{l}{r}}\overset{-1}{\underset{l}{C}}r\right)}^{\text{2}}}{1^{T}\overset{-1}{\underset{l}{C}}1}\right] \end{array}\right. \end{array}$$ (16)

$$\begin{array}{c} \left\{ \begin{array}{c} @c@{\hat{\mu }}_{l}=\frac{1^{T}\overset{-1}{\underset{l}{C}}y_{l}}{1^{T}\overset{-1}{\underset{l}{C}}1}\\ \overset{\text{2}}{\underset{l}{\hat{\sigma }}}=\frac{{\left(y_{l}-1{\hat{\mu }}_{l}\right)}^{T}\overset{-1}{\underset{l}{C}}\left(y_{l}-1{\hat{\mu }}_{l}\right)}{n} \end{array}\right. \end{array}$$ (17)

where ${\hat{y}}_{l}$ denotes the unbiased estimate of the model at the data point x, ${\hat{\mu }}_{l}$ is the mean of the random process, $r_{l}$ is the covariance matrix between the known data points and the predicted points x, $C_{l}$ is the correlation matrix between the known data points, $y_{l}$ is the vector of known data points, 1 is a constant matrix with a value of 1, $\overset{\text{2}}{\underset{l}{s}}\left(x\right)$ is the mean square deviation of the predicted values, which is also used to represent the uncertainty of the Kriging model, and $\overset{\text{2}}{\underset{l}{\hat{\sigma }}}$ is the variance of the random process.

The application of scaling factor $\beta_{\text{0}}$ $\beta_{\text{0}}$ to the low-fidelity Kriging model establishes a global trend, whereas the high-fidelity function is assumed to follow a stochastic process.


$$\begin{array}{c} Y\left(x\right)=\beta_{\text{0}}{\hat{y}}_{lf}\left(x\right)+Z\left(x\right) \end{array}$$ (18)

where $\beta_{\text{0}}$ is a constant that represents the degree of matching between the low-fidelity Kriging function and the high-fidelity function, ${\hat{y}}_{lf}\left(x\right)$ is a random process with a mean of zero, and denotes the predicted value of the low-fidelity Kriging at x.

Consequently, the low-fidelity function was mapped onto a high-fidelity function. Subsequently, an HK model was constructed using a high-fidelity sample datasets $\left(X_{h},y_{h}\right)$. In the context of unbiased conditions, the prediction values for the high-fidelity function at an untested x can be obtained by minimizing the mean-square error (MSE), as shown in (19)–(24).


$$\begin{array}{c} \hat{y}\left(x\right)=\beta_{\text{0}}{\hat{y}}_{l}\left(x\right)+r^{T}\left(x\right)R^{-1}\left(y_{h}-\beta_{\text{0}}F\right) \end{array}$$ (19)

$$\begin{array}{c} \beta_{\text{0}}={\left(F^{T}R^{-1}F\right)}^{-1}F^{T}R^{-1}y_{h} \end{array}$$ (20)

$$\begin{array}{c} F={\left[{\hat{y}}_{l}\left(x^{\text{1}}\right),{\hat{y}}_{l}\left(x^{\text{2}}\right)\text{,...,}{\hat{y}}_{l}\left(x^{n}\right)\right]}^{T} \end{array}$$ (21)

$$\begin{array}{c} R={\left(R\left(x^{i},x^{j}\right)\right)}_{i,j}\in {ℜ}^{n\times n}\mathbb{R}\mathbb{R}\mathbb{R}\mathbb{R}\mathbb{R}\mathbb{R}\mathbb{R}\mathbb{C}\mathbb{R} \end{array}$$ (22)

$$\begin{array}{c} r=R{\left(x^{i},x\right)}_{i}\in \mathbb{R}{ℜ}^{n} \end{array}$$ (23)

$$\begin{array}{c} \begin{array}{c} MSE\left[\hat{y}\left(x\right)\right]=s^{\text{2}}\left(x\right)\\ =\sigma^{\text{2}}\left[\begin{array}{c} @l@1-r^{T}R^{-1}r\\ +\frac{{\left(r^{T}R^{-1}F-{\hat{y}}_{lf}\left(x\right)\right)}^{\text{2}}}{F^{T}R^{-1}F} \end{array}\right] \end{array} \end{array}$$ (24)

#### C. Multi-level expect improvement method.

The advent of technological advancements has precipitated the evolution of surrogate models from their inception as surrogate models through the implementation of various optimization algorithms. These models have been metamorphosed into data-driven sample point addition and optimization mechanisms that approximate local or global optimal solutions, a phenomenon referred to as surrogate-based optimization (SBO) [28]. Mockus et al. [32] proposed a sample encryption criterion called expected improvement (EI), which evaluates and introduces new samples during model training, with the last introduced sample point being the optimal point. Jones et al. [33] combined the Kriging model with EI to establish an efficient global optimization method (EGO). The predicted value of the Kriging model at point ***x*** follows a normal distribution ($Y\left(x\right)\sim \;N\left(\hat{y}\left(x\right),s^{\text{2}}\left(x\right)\right)$). At this juncture, the current optimal predicted value is $y_{min}$, and the decision and expected value at the sample point can be constructed. Proposed by Zhang et al. [7], multilevel EI is shown in (25) and (26). The model fidelity level variable was introduced to determine the sampling location and the fidelity level of the next sampling point. Consequently, the optimization process can automatically converge to the global optimum of the high-fidelity model.


$$\begin{array}{c} s^{\text{2}}\left(x,l\right)=\left\{ \begin{array}{c} @l@\begin{array}{c} @l\overset{\text{2}}{\underset{\text{0}}{\beta }}s^{\text{2}}\left(x\right),\;l=1 \end{array}\\ \begin{array}{c} @ls^{\text{2}}\left(x\right),\;l=2 \end{array} \end{array}\right. \end{array}$$ (25)

$$\begin{array}{c} EI_{vf}\left(x,l\right)=\left\{ \begin{array}{c} @l@\begin{array}{c} @l\begin{array}{c} @l\left(y_{min}-\hat{y}\left(x\right)\right)\phi \left(\frac{y_{min}-\hat{y}\left(x\right)}{s\left(x,l\right)}\right)\\ +s\left(x\right)\varphi \left(\frac{y_{min}-\hat{y}\left(x\right)}{s\left(x,l\right)}\right), \end{array}s\left(x,l\right)>0 \end{array}\\ \begin{array}{c} @l\begin{array}{c} @l\begin{array}{c} @l\begin{array}{c} @l\;0 \end{array}\begin{array}{c} @l \end{array} \end{array} \end{array}\;,\;s\left(x,l\right)=0 \end{array} \end{array}\right. \end{array}$$ (26)

where ϕ and φ denote the cumulative distribution function and probability density function of the standard normal distribution, respectively; $y_{min}$ is the current optimal value within each cycle; $\hat{y}\left(x\right)$ is the predicted value of the Kriging model; s(x) is the root mean square error; *l* is the fidelity level variable; and 1 and 2 represent high-and low-fidelity, respectively.

#### D. KEHK optimization framework.

A flowchart of the proposed KEHK framework for wind tunnel structure optimization is shown in Fig 4. First, the critical design variables were identified via a parameter sensitivity analysis. Second, the knowledge-embedded sequential sampling was carried out started from OLHS. Two finite element models with distinct accuracy levels were established to generate training datasets with different fidelity through a structural response analysis. Then, a primary Kriging surrogate model was constructed using low-fidelity data as the global trend, which was subsequently enhanced through limited high-fidelity samples. Based on the multi-fidelity EI criterion, the hierarchical model automatically undergoes iterative refinement via the addition of new sampling points. Following a thorough evaluation of the sequence sampling, if the trigger output level was 1, a low-fidelity FEA simulation was used to expand the baseline datasets; otherwise, a high-fidelity sample was added. This HK model adaptive-update process continues until convergence, with the final design parameters normalized according to the engineering specifications.

### A. Preliminary routine structural design

**Fig 5:**
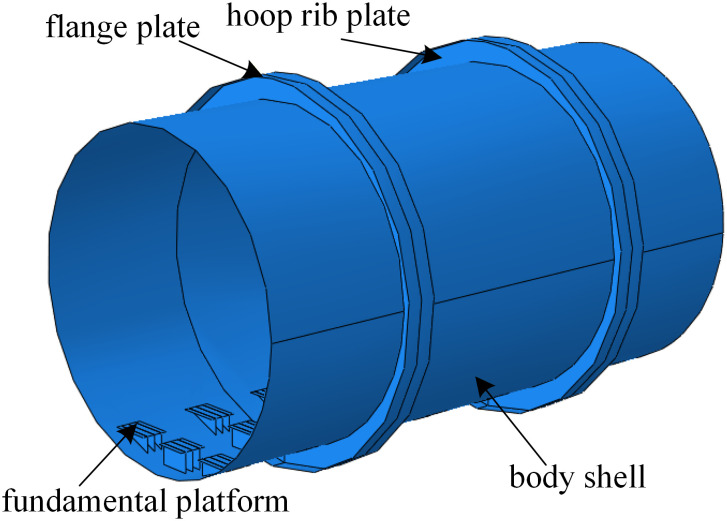
Wind tunnel acceleration section.

The typical structure investigated in this study was the acceleration section of a transonic wind tunnel. Its internal cross section is Ø6 m, with two hoop ribs and flange plates stiffened outside (as shown in Fig 5). The section must sustain an internal pressure of 0.2 MPa and external pressure of 0.1 MPa. To satisfy the flow quality requirement, the total deformation of the cross section should not exceed 0.1%, that is, 6 mm. To ensure the precision of the internal mounting mechanism, it is imperative to restrict the maximum deformation of the fundamental platform to a reasonable range. Furthermore, the first-order intrinsic frequency of the structure, the axial deformation of the section, and other parameters must be considered during the structural design of the wind tunnel.

The design parameters to be determined for the chosen wind tunnel structure design include the material thickness *δ*, number of hoop rib plates and flange plates *n*, height of the hoop rib plates *H*, width of the flange plates *W*, and spacing of the hoop rib plates *L*. In industrial applications, a uniform wall thickness is typically set for sections with similar dimensions to balance the contradiction between optimal technological and engineering management solutions. Thus, shell wall thickness was not set as a design variable in the literature [23]. In fact, plate thickness significantly influences the structure weight; therefore, different wall thicknesses should be selected at the preliminary design stage according to the loads imposed on different areas of the structure. For the accelerated section studied, the material thickness values can be divided into two groups: the cavern body pressure-bearing shell *δ*_*1*_ and hoop rib and flange plates *δ*_*2*_. The primary design variables are listed in Table 1.

**Table 1:** Significance of the design variables.

Design variables	Significance
*n*	number of hoop rib plates
*L*	gap distance of hoop rib plates
*W*	width of flange plates
*H*	height of hoop rib plates
*δ*_*1*_	thickness of body shell
*δ*_*2*_	thickness of hoop rib plates and flange plates

In summary, the typical structural optimization design of a wind tunnel can be abstracted as the constrained optimization problem described in (27)–(31). The optimization objective is the weight of the structure, denoted as M. The constraints include the first-order intrinsic frequency *f*, maximum von Mises stress *S*, axial deformation of the shell *U*_*1*_, and vertical deformation of the fundamental platform *U*_*3*_.


$$\begin{array}{c} x=\left(n,L,W,H,\delta_{\text{1}},\delta_{\text{2}}\right) \end{array}$$ (27)

$$\begin{array}{c} y=\left(f,S,U_{\text{1}},U_{\text{3}},M\right) \end{array}$$ (28)

$$\begin{array}{c} x\mathrm{\backslash stackrel}f\to y \end{array}$$ (29)

$$\begin{array}{c} \underset{x}{min}M \end{array}$$ (30)

subject to:


$$\begin{array}{c} f,S,U_{\text{1}},U_{\text{3}} \end{array}$$ (31)

As illustrated in Fig 2, the design flow stipulates that the number of wind tunnel acceleration section hoop rib plates, designated as n, is set to 2, and the material utilized is Q355R. Considering the structural gravity and internal pressure (0.1 MPa), the design parameters of the cave body were initially selected in accordance with the pressure vessel specification [30]. Structural FEA was subsequently conducted to verify the design. As delineated in Table 2, the initial design parameters and material properties are as follows: density is represented by ρ, Young’s modulus is represented by E, Poisson’s ratio is represented by ν, strength is represented by $\sigma_{ul}$, yield stress is represented by $\sigma_{Y}$, and allowable stress is represented by [σ].

**Table 2:** Material properties of the steel plates used for the acceleration section.

Design parameter		Material parameter	
n	2	$\rho \;(Kg/m^{\text{3}})$	7850
*L* (mm)	6000	$E({MP}_{a})$	206
*W* (mm)	400	*v*	0.3
*h* (mm)	700	$\sigma_{ul}({MP}_{a})$	490
*δ*_*1*_ (mm)	26	$\sigma_{Y}({MP}_{a})$	315
*δ*_*2*_ (mm)	26	$\left[\sigma ]({MP}_{a}\right)$	185

**Fig 6:**
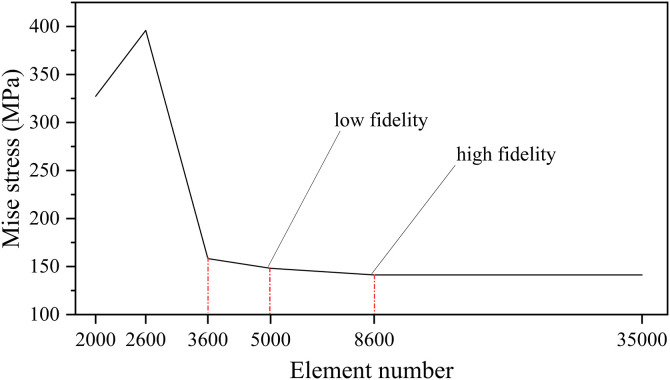
Mesh independence verification.

Mesh independence verification is a critical prerequisite in FEA to eliminate discretization-induced numerical artifacts. This validation process involved systematic mesh refinement under specified operational conditions, with solution convergence serving as the determinant of the optimal element density. Following the industrial standards [34], the relative error threshold between successive refinements was maintained below 5% for solution acceptance. For the structural stiffness assessment, S4R shell elements were implemented throughout this investigation. Fig 6 shows the convergence behavior of the maximum structural von Mises stress ($\sigma_{max}$) versus mesh density.

Fig 5 shows the asymptotic convergence of the maximum von Mises stress ($\sigma_{max}$) beyond 3,600 elements, with the relative variation diminishing below 4.8% at a resolution of 5,000 elements, which satisfies the FEA convergence criteria [34]. The solution invariance threshold emerged at 8,600 elements, establishing this as a high-fidelity benchmark. Computational economy considerations dictated the selection of 5,000-element low-fidelity and 8,600-element high-fidelity models for the multiresolution analysis.

**Fig 7:**
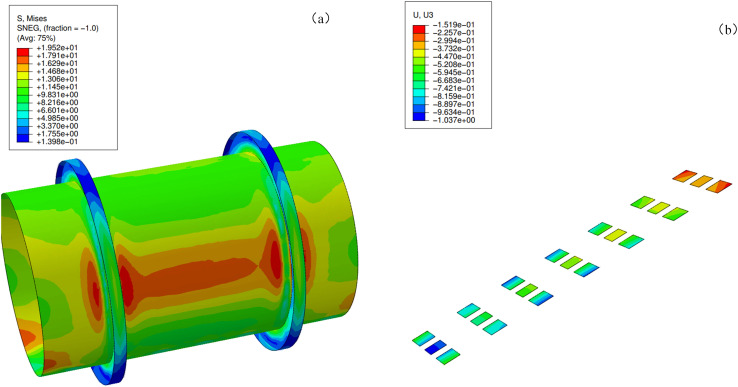
Stress and deformation contours of the primary design. (a) stress contour of the acceleration section. (b) vertical deformation contour of the acceleration section.

Preliminary structural evaluation via the high-fidelity model yielded a fundamental first-order natural frequency of 3.5 Hz with a corresponding $\sigma_{max}$ of 19.5 MPa. The deformation characteristics included 5.8 mm axial displacement and a 1.0 mm vertical platform deflection under operational loading. The integrated system mass totaled 51.9 metric tons, with complete stress–‒strain distributions detailed in Fig 7.

### B. Parameter sensitivity analysis

Sensitivity analysis (SA) serves as a fundamental methodology in engineering design for quantifying the uncertainty propagation from input parameters to system outputs [35–37]. This technique achieves model simplification by probabilistically treating high-impact variables while fixing low-sensitivity parameters as deterministic constants, thereby optimizing structural performance under economic constraints [38]. The framework encompasses two complementary approaches: local sensitivity analysis (LSA), which examines perturbation effects around nominal values; and global sensitivity analysis (GSA), which evaluates parameter interactions across complete operational ranges.

**Fig 8:**
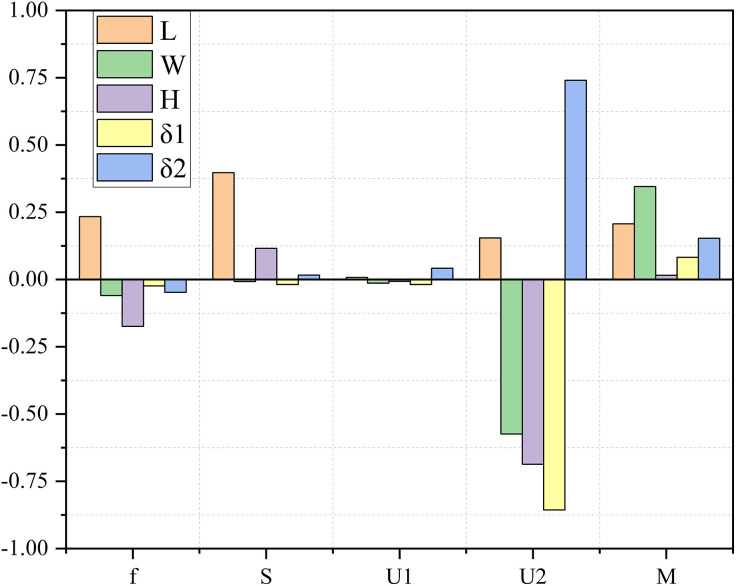
Parameter sensitivity analysis.

The study employs Pearson correlation coefficients [39] for parameter sensitivity analysis, with the variable response relationships visualized in Fig 8. A total of 300 sample datasets were used for the analysis. Positive values indicate that the optimization objective or constraint increases as the variable value increases, referred to as positive correlation; negative values indicate that the optimization objective or constraint decreases as the variable value increases, referred to as negative correlation. The first-order natural frequency demonstrates the primary sensitivity to the flange width W, followed by comparable influences from the reinforcement span L and secondary plate thickness δ_2_, whereas the primary cylinder thickness δ_1_ has a moderate correlation, and the flange height H has a negligible impact. Notably, all of these factors were positively correlated. The maximum von Mises stress reveals contrasting thickness dependencies, with δ_1_ displaying an inverse correlation relative to δ_2_ and the former presenting the greatest significance. The axial deformation U_1_ of the structure has a discernible negative correlation with δ_1_, whereas L and W have comparable effects on the theoretical directions. The fundamental plate deformation U_3_ and mass M both demonstrate thickness-dominated responses, with the primary influencing factor being δ_1_, resulting in differential correlation patterns.

In the context of engineering applications, the natural frequency is not only influenced by design variables but also highly sensitive to structural installation methods and support configurations. U_1_ is approximately linearly proportional to the linear expansion coefficient of the material, the structural length, and the temperature difference. Consequently, the effects of *f* and U_1_ were not addressed in this study. Consequently, the optimization framework selects L, W, δ_1_, and δ_2_ as design variables, constrained by the peak stress S and platform displacement U, while minimizing the structural mass M. This systematic formulation transforms the original design problem of (27)–(31) into a computationally tractable optimization system of (32)–(36) through constraint prioritization and variable screening.


$$\begin{array}{c} x=\left(L,W,\delta_{\text{1}},\delta_{\text{2}}\right) \end{array}$$ (32)

$$\begin{array}{c} y=\left(S,U,M\right) \end{array}$$ (33)

$$\begin{array}{c} \underset{x}{min}M \end{array}$$ (34)

subject to:


$$\begin{array}{c} S<185 \end{array}$$ (35)

$$\begin{array}{c} U\leq 2 \end{array}$$ (36)

where S is the maximum von Mises stress (MPa), U is the vertical displacement of the fundamental platform (mm), and M is the total structural mass (tons).

### C. Wind tunnel structure optimization

In the context of optimization algorithms, constraints are often converted into unconstrained optimization problems through the application of equivalent methods. The fundamental principle underlying the EI optimization algorithm is to attain the desired final optimization objective by ascertaining the direction of expectation maximization. When combined with genetic algorithms, the search is minimized by taking the opposite value of the expectation amplitude. Consequently, the constrained optimization problem described in (32)–(36) is expressed in the form of (37)–(39).


$$\begin{array}{c} \Gamma \left(x\right)=y_{\text{3}}\left(x\right)+p_{\text{1}}\left(y_{\text{1}}\right)+p_{\text{2}}\left(y_{\text{2}}\right) \end{array}$$ (37)

$$\begin{array}{c} p_{\text{1}}\left(y_{\text{1}}\right)=\left\{ \begin{array}{c} @l\begin{array}{c} 0,\;y_{\text{1}}\;<185 \end{array}\\ \begin{array}{c} @l1e5,\;y_{\text{1}}\;\geq 185 \end{array} \end{array}\right. \end{array}$$ (38)

$$\begin{array}{c} p_{\text{2}}\left(y_{\text{2}}\right)=\left\{ \begin{array}{c} @l\begin{array}{c} @l0,\;y_{\text{2}}\;<5 \end{array}\\ \;\;\;\;\begin{array}{c} 1e5,\;y_{\text{2}}\;\geq 5 \end{array} \end{array}\right. \end{array}$$ (39)

Based on engineering experience, the thickness of the body shell should not be less than that of the hoop rib and flange plates. Then, (20) can be transformed into as (40)–(41).


$$\begin{array}{c} \delta_{\text{2}}=S_{n2}(\delta_{max}-\delta )+\delta \end{array}$$ (40)

$$\begin{array}{c} \delta_{\text{1}}=S_{n1}(\delta_{max}-\delta_{\text{2}})+\delta_{\text{2}} \end{array}$$ (41)

where $\delta_{max}$ represents the maximum permissible thickness, measured in millimeters, and $S_{n1}$ and $S_{n2}$ denote the optimal Latin hypercube sampling parameters.

Based on the findings of the parameter sensitivity analysis, the reinforcement plate height was set to 500 mm. Additionally, 160 and 10 low-fidelity samples were used for the respective analyses. Equations (40) and (41) ensure compliance of the material thickness with design specifications. The function variable in (26) is identified as the material thickness, as shown in (42). The knowledge-embedded sequential sampling process was implemented using MATLAB. The minimum geometric distance between the initial sample points was adopted as the threshold for the distance-based judgment. A constrained optimization problem involving three objective parameters and four design variables for the wind tunnel acceleration section was validated. By integrating MATLAB, Python, and ABAQUS, an adaptive structural optimization design framework was developed and automated.


$$\begin{array}{c} \begin{array}{c} f\left(L_{s}\right)=\frac{0.55\sqrt{D\delta }D^{\text{3}}+\delta {\left(D+2h\right)}^{\text{3}}}{12}\\ -\frac{D^{\text{2}}\left(4L_{s}\delta +\pi \left({\left(D+2h\right)}^{\text{2}}-D^{\text{2}}\right)\right)}{43.6}A\geq 0 \end{array} \end{array}$$ (42)

**Fig 9:**
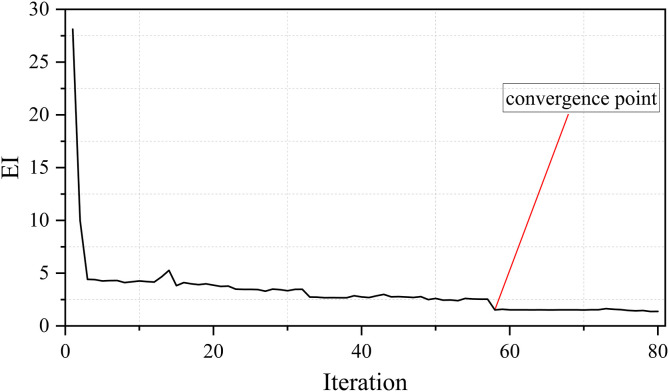
Optimization process of the structural EI.

A preliminary HK surrogate model was initially constructed using 160 low-fidelity samples and 10 high-fidelity samples, with hyperparameter tuning performed using a genetic algorithm described in the literature [40]. A total of 80 iterations were set as the maximum, and the optimal value of EI at each optimization iteration step is illustrated in Fig 9.

As shown in Fig 9, after 52 iterations, the structural mass reached a stable optimized weight of 35.2 tons. The optimal design parameters are listed in Table 3. At this stage, 11 high-fidelity samples and 40 low-fidelity sample points were incorporated.

**Table 3:** Optimum structural design parameters.

Design Parameter	L(mm)	W(mm)	δ_1_(mm)	δ_2_(mm)
Primary value/mm	6570	415	18.7	13.4
Uniformed value/mm	6600	420	20	16

**Fig 10:**
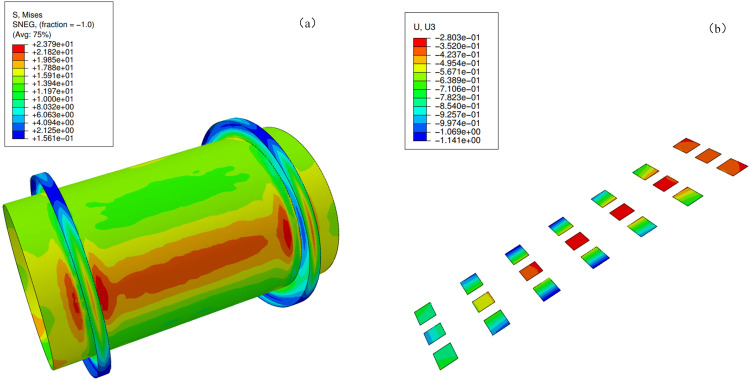
The stress and deformation contours of the optimized design. (a) the stress contour of the acceleration section. (b) the vertical deformation contour of the acceleration section.

In engineering practice, material thickness selection adheres to a standardized dimension series, including 6, 8, 10, 12, 16, 20, 26, 30, 32, 36, and 40 mm. Flange plates and hoop rib plates are typically fabricated via on-site welding processes characterized by limited precision, with a tolerance margin of approximately 10 mm. Consequently, the flange plate width was generally specified as a factor of 10 mm, whereas the hoop rib plate spacing was 100 mm. To ensure structural safety compliance, the upward rounding principle was uniformly applied when standardizing wall thickness specifications. Following parameter normalization, as detailed in Table 3, the optimized design yielded a total weight of 38.4 tons, a principal stress of 23.8 MPa, and a maximum vertical displacement of 1.1 mm for the support platform. The corresponding stress distribution contours are shown in Fig 10.

The surrogate model demonstrated significant computational efficiency, reducing the simulation time from 60.4 s per high-fidelity FEA to 0.4 s per prediction, a 150-fold increase in computational efficiency has been achieved. Compared with conventional finite-element-based empirical optimization, this adaptive parameter search methodology significantly enhances the overall optimization efficiency while maintaining the design integrity. All coding and FEA simulations in this study were performed on a computer equipped with 3th Gen Intel Core i9-13900K platform with 128 GB of RAM and an NVIDIA RTX 4080 GPU.

To validate the effectiveness of the proposed KEHK framework, comparative optimization experiments were performed on the structures described in Section Ⅲ using both back-propagation neural networks (BPNN) and response surface methodology (RSM). The genetic algorithm (GA) served as the optimization algorithm for both approaches.

BPNN comprises three hidden layers containing 100, 200, and 100 neurons, respectively. For the GA, the population size is set to 30, the crossover rate to 0.8, the mutation rate to 0.25, and the convergence criterion to 10 ⁻ ⁶. Identical GA parameter settings are adopted for both the BPNN and the RSM optimization. The maximum number of iterations is capped at 100 for both surrogate-based approaches; the BPNN and RSM models converge after 79 and 93 iterations, respectively—comparable to the convergence behavior observed with the KEHK method. Following optimization, the resulting structural weights are 40.5 t (BPNN) and 41.2 t (RSM), both satisfying all prescribed design constraints. Comparative results are summarized in Table 4. As both the BPNN and RSM represent single-fidelity surrogate modeling strategies, their training datasets consist of a hybrid set comprising 160 low-fidelity samples and 10 high-fidelity samples.

**Table 4:** Comparison table of different surrogate models.

Model	Parameter	Optimization algorithm	Dataset	Iteration	Mass(ton)
KEHK	Knowledge-embedded hierarchical Kriging	GA	high fidelity 160 low fidelity 10	52	35.8
BPNN	100，200，100	GA	160 + 10	79	40.5
RSM	3 order	GA	160 + 10	93	41.2

As shown in Table 4, the optimal structural weights obtained via BPNN and RSM are 5.59% and 7.18% higher than KEHK method while satisfying the maximum allowable stress and deformation constraints on the support platform. This suggests that the single-fidelity surrogates may have converged to local optima rather than the global optimum. In contrast, the proposed KEHK framework explicitly incorporates prior engineering knowledge thereby yielding optimization outcomes that better align with practical engineering requirements.

## Discussion

### A. Model generalization performance research

Surrogate models generally demonstrate excellent predictive capabilities within the confines of the training datasets. Nevertheless, their capacity to predict operating conditions that fall outside the boundaries of the training datasets — that is to say, the generalization performance of the model — is of paramount importance for the broader utilization of the model. For the proposed KEHK model, HK described in literature [41] and the BPNN were selected to conduct a comparative validation of the model’s generalizability across the transonic wind tunnel operating range.

For transonic wind tunnels, the standard operating parameters that are typically employed encompass pressure, temperature, and gas velocity. As previously discussed, the present study exclusively considers the wind tunnel’s response to static loads; consequently, the gas velocity is not factored into the analysis at this stage. Additionally, for the majority of transonic wind tunnels, due to their open layout, variations in tunnel temperature are negligible and can be disregarded in this study. However, it is important to note that when the structure is employed in a continuous-flow wind tunnel, temperature variations in the tunnel caused by factors such as the continuous operation of the power system are significant and cannot be ignored. In summary, this comparison of model versatility across operating conditions will temporarily consider only the influence of pressure. The maximum pressure provided by the air supply system of a transonic wind tunnel is typically 2.0 MPa; therefore, the operating pressure range is generally between 0.1 and 2.0 MPa.

As outlined in Section Ⅱ, wind tunnel operating conditions were not directly factored into the KEHK model training process. However, during the sequence sampling stage, the parameter generation for wall thickness incorporates the influence of pressure.

The KEHK model incorporates the influence of the operating pressure during the calculation of the material plate thickness through the embedding of design specifications via (1). The dimensions L, W, and H were held constant in accordance with the optimal design outlined in the preceding section. The minimum wall thickness, as determined by (1), was calculated under the specified operating conditions. In accordance with the sequence of plate thickness values stipulated by the engineering material, the material wall thickness value corresponding to the verification operating conditions was obtained according to the upward rounding principle. The corresponding sample sampling coefficient is subsequently solved using (45) and (46) and substituted into the surrogate model to obtain the corresponding operating condition prediction values. This method of directly calculating the wall thickness for each verification condition and invoking the surrogate model can be referred to as the normal KEHK.


$$\begin{array}{c} \overset{i}{\underset{n2}{S}}=\frac{\overset{i}{\underset{\text{2}}{\delta }}-\delta^{i}}{\delta_{max}-\delta^{i}} \end{array}$$ (45)

$$\begin{array}{c} \overset{i}{\underset{n1}{S}}=\frac{\overset{i}{\underset{\text{1}}{\delta }}-\overset{i}{\underset{\text{2}}{\delta }}}{\delta_{max}-\overset{i}{\underset{\text{2}}{\delta }}} \end{array}$$ (46)

where $\overset{i}{\underset{\text{1}}{\delta }}$, $\overset{i}{\underset{\text{2}}{\delta }}$ denotes the actual values of $\delta_{\text{1}}$ and $\delta_{\text{2}}$ for validation condition *i*; $\delta^{i}$ denotes the corresponding calculated thickness; and $\overset{i}{\underset{n1}{S}}$ and $\overset{i}{\underset{n2}{S}}$ denote the sampling values.

As illustrated in the surrogate model training process described above, all samples were generated based on the training conditions, and the corresponding material thickness selection was based on the minimum calculated wall thickness δ under these conditions. To maximize the advantages of embedded knowledge, constructing a conversion relationship between the verification and training conditions is more reasonable. The converted parameters can then be used to drive the surrogate model to perform structural von Mises prediction. As demonstrated in (47) and (48), under the verification conditions, the calculated wall thickness δ of the training conditions was designated as the minimum wall thickness, and the conversion sampling coefficient corresponding to the verification conditions was solved to drive the surrogate model to predict the results. This process is referred to as condition mapping KEHK.

In the two aforementioned methods, when the corresponding wall thickness fails to meet the FEA convergence requirements, the material wall thickness is reselected to the previous specification.


$$\begin{array}{c} \overset{j}{\underset{n2}{S}}=\frac{\overset{j}{\underset{\text{2}}{\delta }}-\delta }{\delta_{max}-\delta } \end{array}$$ (47)

$$\begin{array}{c} \overset{j}{\underset{n1}{S}}=\frac{\overset{j}{\underset{\text{1}}{\delta }}-\overset{j}{\underset{\text{2}}{\delta }}}{\delta_{max}-\overset{j}{\underset{\text{2}}{\delta }}} \end{array}$$ (48)

where $\overset{j}{\underset{\text{1}}{\delta }}$, $\overset{j}{\underset{\text{2}}{\delta }}$ denotes the actual value of $\delta_{\text{1}}$ and $\delta_{\text{2}}$ for validation condition *j*; $\overset{j}{\underset{n1}{S}}$ and $\overset{j}{\underset{n2}{S}}$ denotes the corresponding sampling value, and δ denotes the calculated thickness of the training condition.

**Fig 11:**
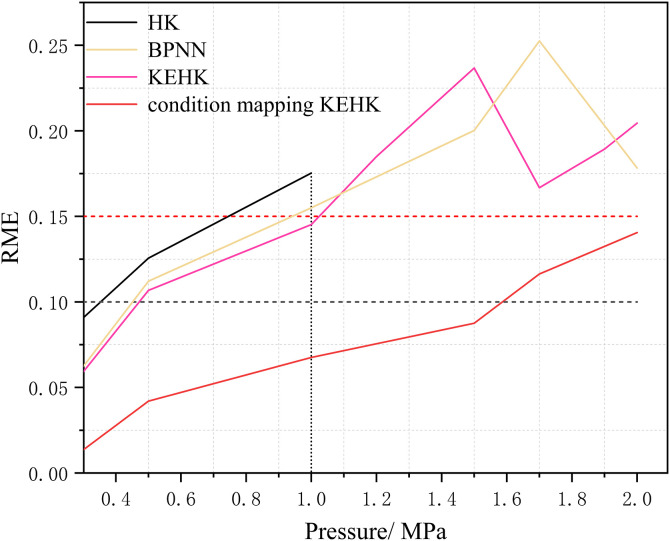
Relative error curve diagram.

As demonstrated in Fig 11, under the conditions of validation, the discrepancy between the maximum Von Mises and FEM result distributions for the HK, BPNN, KEHK and conditional KEHK models is exhibited. For the HK model, the validation threshold settings indicated that subsequent operating conditions were no longer displayed when the relative error exceeded 15% at 1.0 MPa. The HK model demonstrates the poorest generalization performance, with a relative error that exceeds 10% when the operating pressure increases by 0.3 MPa. A comparative analysis of the BPNN and KEHK models reveals that they demonstrate marginal superiority over the HK model with regard to generalizability. Within the range of 0.1–1.1 MPa, the prediction error of the BPNN model was marginally higher than that of the KEHK model. Among the four models, the conditional mapping KEHK model demonstrated the best generalization performance, attaining effective prediction results across the entire range of validation operating conditions with a maximum prediction error of 14.1%. Despite an increase in the operating pressure to 1.6 MPa, the prediction error remained at a slightly elevated level, remaining above 10%.

As demonstrated by the comprehensive analysis, embedded knowledge can significantly enhance the model generalization performance in comparison with the pure data-driven HK model. Following the validation process, which was based on the conversion of the operating conditions, the proposed KEHK model demonstrated excellent performance prediction and superior generalization application capabilities within the conventional continuous transonic wind tunnel operating pressure range. These results indicate significant potential for engineering applications and promotion of the proposed model.

### B. Initial training sample size effects

The determination of the initial datasets size represents a critical aspect in optimizing multi-fidelity surrogate models. The literature [18] establishes that multi-fidelity Kriging models require initial high-fidelity and low-fidelity sample quantities proportional to the number of design variables m. The minimum low-fidelity sample size is derived from 25(m-1), whereas the high-fidelity samples follow conditional assignments: 10 samples for m < 5 and 20 samples for m ≥ 5. The application of these criteria yielded an initial sample counts of 10/75. Reference [16] optimized a metal column shell via a multi-fidelity surrogate model and revealed the distinct behavioral thresholds as follows: the model accuracy improves inversely with the ratio of low-fidelity samples to high-fidelity samples when it is less than eight but decreases proportionally when it exceeds this threshold. Based on these findings, high- and low-fidelity samples were initialized at 10 and 80, respectively. Model validation employed the root mean squared error (RMSE) and normalized root mean square deviation (NRMSD) formally defined in (43) and (44), with the baseline metrics documented in Table 5.

**Table 5:** Initial sample size training accuracy.

*n_l*	*n_h*	RMSE			NRMSD		
		*U*	*S*	*M*	*U*	*S*	*M*
80	10	0.13	9.43	1.29	0.37	0.46	0.0057


$$\begin{array}{c} RMSE=\sqrt{\frac{\overset{n}{\underset{i=0}{\sum }}{\left(\overset{\left(i\right)}{\underset{real}{y}}-{\hat{y}}^{\left(i\right)}\right)}^{\text{2}}}{n}} \end{array}$$ (43)

$$\begin{array}{c} NRMSD=\frac{RMSE}{{\overset{―}{y}}_{real}}\times 100\% \end{array}$$ (44)

where $y_{real}$ and $\hat{y}$ denote the training value (simulation or experiment) and model-predicted value, respectively, ${\overset{―}{y}}_{real}$ denotes the mean value of $y_{real}$; and n denotes the number of training samples.

**Fig 12:**
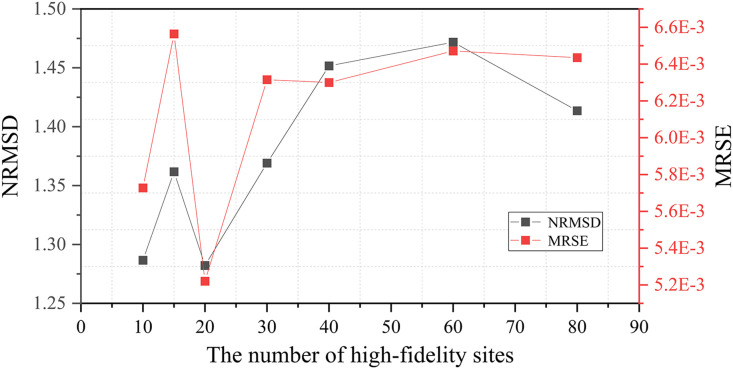
Model accuracy curve with increasing number of high-fidelity samples when the number of low-fidelity samples is 80.

The changes in the RMSE and NRMSD with increasing sample size when the number of low-fidelity samples is fixed at 80 and the number of high-fidelity samples is gradually increased are shown in Fig 12. As illustrated in the figure, there appears to be no discernible trend of enhanced model accuracy with an increasing sample size. Therefore, it was preliminarily determined that the number of low-fidelity samples was significantly smaller than the actual requirement.

While the number of high-fidelity samples was maintained at 10, the number of low-fidelity samples was gradually increased. The results of this study are summarized in Table 6. The findings suggest a direct correlation between the increase in the number of low-fidelity samples and the rapid decline in the predicted NRMSD values and RMSE for structural quality M. Notably, when the number of low-fidelity samples exceeds 160, the NRMSD value decreases by a factor of magnitude of 10 ⁻ ⁴. Furthermore, as the number of samples increases, the rate of decrease in both metrics slows significantly, indicating that the accuracy reaches a state of stability. Consequently, the number of low-fidelity samples was determined as 160.

**Table 6:** Model accuracy with 10 high-fidelity samples.

Sample size	RMSE	NRMSD
80	1.29	5.73 × 10^−3^
100	0.59	2.62 × 10^−3^
160	0.21	9.29 × 10^−4^
200	0.18	8.02 × 10^−4^

As demonstrated by the findings of the aforementioned research, the results are consistent with those in [18]. For the four-variable, three-objective constraint optimization problem in this study, it is appropriate to set the number of high-fidelity samples to 10 when training the HK surrogate model. However, the study did not identify a significant correlation between the model accuracy and the ratio of different fidelity sample numbers, which does not align with the conclusions reported in [16]. The study in [16] was based on the Co-Kriging model, which differs from the HK model used in this study. The distinguishing aspect between this study and the research outlined in the literature [16] is that the former calculates the yield stress of a composite cylinder, which is the result of the combined effect of various design variables. In this study, while the maximum stress and displacement constraints were also influenced by various structural parameters, the object of study was the von Mises stress. The indicators directly related to the structural parameters were the stress components. Following the execution of the synthesis calculations, the relationship between the von Mises stress and the design variables becomes more implicit. Furthermore, as the design variables are altered, the location of the maximum von Mises stress may also change. This also implies that the position variable of the maximum stress is implicitly included in the optimization process. As shown in Table 6, among the three output variables, the RMSE and NRMSD related to S are the most substantial.

## Conclusion

In this study, a knowledge-embedded sequential sampling method is proposed, which combines the normal OLHS and pressure vessel design specifications. Based on an adaptive surrogate model, a framework for the structural optimization design of typical wind tunnel pressure vessel structures was established, with a focus on integrating prior knowledge to increase model accuracy and generalization performance. This framework incorporates pressure vessel specifications through systematic dataset sampling and generation. A hierarchical Kriging interpolation model was established using multi-fidelity training data to predict the structural responses. Structural optimization is subsequently achieved by employing a multi-fidelity expected improvement criterion, which adaptively judges and continuously introduces new sample points at distinct fidelity levels. Finally, the most recently introduced sample points are normalized based on engineering practice experience.

To validate the proposed method, a structural optimization case study of a transonic wind tunnel acceleration section (internal diameter of 6 m) was conducted. The impact of the initial training sample size on the model prediction accuracy was analyzed, and the generalization performance of the proposed model was verified across the operational conditions of a transonic wind tunnel. Our findings can be summarized as follows:

After 52 iterations, the acceleration section of the transonic wind tunnel achieves a typical weight reduction of approximately 26%. The single prediction response time of the surrogate model was approximately 0.6% of that of FEA, representing a significant improvement in computational efficiency while maintaining the prediction accuracy.

Comparative studies confirmed that the initial selection of high-fidelity samples aligns with the results from the literature.

The proposed parameter mapping methodology, validated within the 0.1–2.0 MPa operating pressure range of transonic wind tunnels, demonstrated the advantages of the KEHK model in terms of prediction accuracy and applicability across operating conditions. These results underscore the excellent generalization performance and strong engineering potential of this method.

As previously discussed in this paper, wind tunnels are exposed to a multitude of dynamic and static loads during operation, encompassing aerodynamic, mechanical, and thermodynamic forces. The accurate prediction of structural responses under all operating conditions remains a key focus in the fields of wind tunnel design and operation and maintenance. For future work, the primary objective is to incorporate additional load types into response analysis, with a particular focus on dynamic loads such as airflow pulsations and vibrations. This will enable the acquisition of more precise response predictions. Secondly, the incorporation of supplementary design specifications into the surrogate optimization framework, in conjunction with the expansion of the scope of structural design variables in the optimization process, is investigated to enhance the engineering applicability of the method and improve wind tunnel structural design efficiency. Conducting sensitivity analyses is imperative to assess the framework’s response to inaccuracies or uncertainties within these prior knowledge constraints. Thirdly, the scope of operating condition parameters within the framework must be expanded to include extreme pressures, temperatures, and velocities, thereby enabling application coverage ranging from conventional wind tunnels to specialized facilities such as high-temperature, hypersonic, and cryogenic wind tunnels. Furthermore, the framework should be applicable to the digital and intelligent design and operation of similar equipment, including industrial pressure pipelines, ships, aerospace thin-walled structures, and high-pressure gas storage and transportation systems.
